# Cytotoxicity of Typical Diiodoalkanes from Shale Gas Wastewater in HepG2 Cells

**DOI:** 10.3390/toxics13110943

**Published:** 2025-10-31

**Authors:** Maoyuan Xu, Yusheng Wu, Yunmei Cai, Ruijie Wang, Guofa Ren

**Affiliations:** 1 Institute of Environmental Pollution and Health, School of Environmental and Chemical Engineering, Shanghai University, Shanghai 200444, China; 2 School of Environmental Monitoring, Guangdong Polytechnic of Environmental Protection Engineering, Foshan 528216, China

**Keywords:** shale gas, diiodoalkanes, HepG2 cells, oxidative stress, transcriptomic analysis

## Abstract

Shale gas extraction releases significant quantities of organic iodides of “unknown origin”, which generally pose high ecological and health risks, yet their toxic mechanisms remain unclear. In this study, the human hepatocellular carcinoma (HepG2) cell line was employed as an in vitro cell model to assess the cytotoxic effects of three typical organic iodides (1,2-diiodoethane, 1,3-diiodopropane, and 1,4-diiodobutane) identified in shale gas extraction wastewater from Chongqing, China. The results demonstrated that all three diiodoalkanes exhibited significant toxic effects on HepG2 cells at a concentration of 25 µM, and this effect demonstrated a dose-dependent pattern. As the concentration of diiodoalkanes increased, the viability of HepG2 cells decreased significantly, while cell mortality increased markedly. The transcriptomic analysis indicated that exposure to these three diiodoalkanes induced abnormal expression of genes associated with the extracellular space, extracellular matrix (ECM), and endoplasmic reticulum (ER) in HepG2 cells, which was presumed to be linked to the disruption of the intracellular redox-antioxidant system homeostasis by the diiodoalkanes. Furthermore, assays of intracellular reactive oxygen species (ROS) and antioxidant enzyme/molecule levels suggested that diiodoalkane exposure triggered excessive intracellular ROS production, induced oxidative stress, and ultimately resulted in cell death.

## 1. Introduction

Iodine, as a ubiquitous trace element, has consistently been a research hotspot due to its significance in both biological and geochemical fields [1,2]. The forms of iodine in the environment are diverse. Currently, most research primarily focuses on inorganic iodine, mainly iodide ions (I^−^) and iodate ions (IO_3_^−^), with relatively limited attention paid to iodine-containing disinfection byproducts (I-DBPs) generated during drinking water disinfection [3,4,5]. However, research on other organic iodides in water remains relatively scarce.

The distribution of iodine in the natural environment exhibits significant heterogeneity: apart from marine systems (where seawater contains relatively high iodine concentrations), iodine is also closely associated with natural gas extraction, with shale gas extraction as a typical example [6]. This association leads to sustained elevated iodine levels in the surroundings of shale gas extraction sites. In recent years, halogenated organic compounds (HOCs) have been frequently detected in shale gas wastewater [7,8,9,10]. Notably, several studies have identified organic iodides as the dominant class of detected HOCs. For instance, Luek et al. reported that in flowback wastewater from the Marcellus Shale, organic compounds containing iodine, bromine, chlorine, and mixed halogens (two distinct halogens) accounted for 52%, 20%, 9%, and 19% of total HOCs, respectively [10]. Furthermore, the concentration of organic iodides showed a gradual increase during wastewater discharge and remained relatively high even 10 months after gas well production began [8]. In our previous study, 21 HOCs were identified from the discharged wastewater of the shale gas wastewater treatment plant in Chongqing, China [11]. The composition included 5 chlorinated, 10 iodinated, 3 brominated, and 3 mixed-halogen (bromo/chloro or iodo/chloro) organic compounds. Consistent with prior findings, iodinated organic compounds constituted the most abundant HOC class detected. Quantitative analysis further revealed that diiodoalkanes (including 1,2-diiodoethane, 1,3-diiodopropane, and 1,4-diiodobutane) were present at relatively high concentrations. Among these, 1,2-diiodoethane reached a maximum concentration of 72.6 μg/L (0.26 μM) in surface water [12]. The formation mechanism of organic iodides in shale gas extraction may resemble that of DBPs in drinking water: during hydraulic fracturing or wastewater treatment, dissolved inorganic iodine is oxidized by strong oxidants to generate reactive halogen species (e.g., molecular halogens and halogen-free radicals). These reactive species then react with natural organic matter (NOM) in geological formations to form “unknown-origin” organic iodides [9,11].

HOCs are typically persistent organic pollutants, characterized by chemical stability and low biodegradability, which pose severe threats to the ecological environment and human health. Consequently, HOCs remain a core focus in environmental science. As transformation products of inorganic iodine, organic iodides exhibit far more complex environmental behaviors and toxic effects than their inorganic counterparts. For example, organic iodides generated via atmospheric photochemical processes (e.g., iodomethane and iodoethane) have low intrinsic toxicity but trigger cascading environmental reactions: these volatile compounds undergo long-range atmospheric transport, decompose under ultraviolet (UV) radiation, and release reactive iodine atoms that deplete stratospheric ozone [13]. Additionally, limited cellular and genetic studies on DBPs have demonstrated that brominated and iodinated DBPs exhibit significantly higher cytotoxicity and genotoxicity than their chlorinated congeners [4,14]. Based on these findings, it can be inferred that organic iodides formed during shale gas extraction may also possess substantial toxicity. However, the mechanisms underlying their toxic effects remain poorly understood, and relevant research is extremely limited. Given that shale gas has become a key focus of global energy development, and considering the “high complexity, strong toxicity, and persistence” of organic iodine pollutants derived from shale gas extraction, comprehensive ecological and human health risk assessments prior to shale gas development are of great significance.

Transcriptome analysis unravels the molecular mechanisms by which toxins disrupt cellular gene expression by systematically deciphering dynamic changes in cellular transcriptional states and gene regulatory networks. Within this framework, RNA sequencing (RNA-seq) stands as a core, foundational transcriptomic technology [15]. By leveraging the advantages of high-throughput sequencing, this technique comprehensively captures cellular transcriptomic profiles and precisely identifies differentially expressed genes (DEGs) upon toxic exposure. Upon integration with bioinformatics analyses—such as gene functional annotation and pathway enrichment analysis—RNA-seq can further delineate the mechanisms by which toxic substances perturb core cellular pathways, including those governing metabolism, stress responses, and apoptotic regulation, thereby providing critical molecular insights into the toxic mechanisms of the target substances. Given the high detection frequency and potential high toxicity of iodoalkanes in shale gas wastewater, clarifying the key mechanisms underlying their biological damage is critical. In this study, we evaluated the toxic effects of three typical diiodoalkanes, namely 1,2-diiodoethane, 1,3-diiodopropane, and 1,4-diiodobutane, on HepG2 cells. Transcriptomic analysis was further performed on HepG2 cells exposed to these pollutants, with the aim of identifying key signaling pathways involved in the toxic effects of typical diiodoalkanes.

## 2. Materials and Methods

### 2.1. Chemicals and Reagents

1,2-diiodoethane (C_2_H_4_I_2_, CAS:624-73-7, 98% purity), 1,3-diiodopropan (C_3_H_6_I_2_, CAS:627-31-6, 98% purity), and 1,4-diiodobutane (C_4_H_8_I_2_, CAS:628-21-7, 98% purity) were purchased from Tokyo Chemical Industry Co., Ltd (Tokyo, Japan). The Dulbecco’s modified Eagle medium (High Glucose DMEM) was bought from Datahill Biotechnology Co., Ltd. (Shanghai, China). The Fetal Bovine Serum (FBS) was obtained from Biological Industries Israel Beit Haemek Ltd (Beit Haemek, Israel). Serum-free animal protein-free cell freezing medium was derived from Nell Cell & Molecular Biotech Co., Ltd. (Jiangsu, China). 2′,7′-dichlorofluorescein diacetate (DCFH-DA) and 3-(4,5-dimethylthiazol-2)-2,5-diphenyltetrazolium bromide (MTT) were bought from Sigma-Aldrich (St. Louis, MO, USA). Other unspecified reagents were purchased from Beyotime Biotechnology (Shanghai, China).

### 2.2. Cell Culture

The human hepatocellular carcinoma (HepG2) cell line was purchased from the American Type Culture Collection (ATCC). Cells are routinely cultured in Dulbecco’s Modified Eagle Medium (DMEM) supplemented with 10% fetal bovine serum (FBS) and 1% penicillin-streptomycin (P/S), in a humidified incubator maintained at 37 °C with 5% carbon dioxide (CO_2_). Cells should be passaged at least twice. When cells exhibited stable morphology and reached the logarithmic growth phase (prior to confluence), they were seeded into 96-well or 6-well plates. Following 24 h of adherent culture to allow cell attachment, treatment with diiodoalkanes was initiated. Diiodoalkanes were dissolved in dimethyl sulfoxide (DMSO) for preparation. A solvent control group was included in the experiment, and the final concentration of DMSO in the medium across all groups was maintained at 0.1% to rule out potential solvent-induced interference with cell viability or function.

### 2.3. Cell Viability Assay

MTT assay was employed to evaluate the toxicity and cell viability of diiodoalkanes on HepG2 cells: HepG2 cells were seeded at a density of 4 × 10^3^ cells/well in a 96-well plate. After 24 h of pre-incubation at 37 °C in a humidified incubator with 5% CO_2_ to allow cells to adhere and stabilize, cells were treated with 25 µM, 50 µM, 100 µM, and 200 µM concentrations of diiodoalkanes, and 0.1% DMSO (solvent control). After 24 h exposure, 100 µL of MTT reagent (final concentration ~5 mg/mL) was added to each well and incubated in the dark for 4 h to form MTT crystals. After removing the supernatant, 150 µL of DMSO was added to each well, and the mixture was incubated on a shaking platform in the dark for 3 min to completely dissolve the crystals. Finally, absorbance at 570 nm was measured using a Spark^®^ 20M multimode microplate reader (Tecan, Switzerland). Absorbance values indirectly reflected live cell numbers and cell viability.

### 2.4. Cellular Morphology Analysis

Cells were gently rinsed 1–2 times with phosphate-buffered saline (PBS) to remove residual medium, and the sample was then mounted on the optical microscope stage. During observation, the cell distribution area was first located using the 10× low-power objective, after which the objective was switched to the 20× and 40× medium-to-high power ones. The overall cell morphology was examined and photographed using the microscope’s imaging system, with a focus on recording typical damage features such as cell shrinkage, membrane rupture, abnormal aggregation, or detachment. At least three representative fields per sample were selected to ensure the reliability of the analysis.

### 2.5. RNA-Sequencing and Bioinformatics Analysis

HepG2 cells were exposed to 50 µM 1,2-diiodoethane, 1,3-diiodopropane, and 1,4-diiodobutane for 12 h. Total cellular RNA was then extracted using TRIZOL reagent (Life Technologies, Carlsbad, CA, USA). RNA quality was assessed with an Agilent Bioanalyzer 2100 (Agilent Technologies, Santa Clara, CA, USA) to ensure suitability for subsequent experimental procedures. To guarantee the reliability of results, three biological replicates were included throughout the experiment. The quality assurance and quality control (QA/QC) information for the raw sequencing data and omics analysis of RNA-seq are provided in the Supplementary Materials. cDNA library sequencing was performed by OE Biotech (Shanghai, China) using an Illumina HiSeqTM 2000 sequencer (Illumina, Inc., San Diego, CA, USA). Subsequently, DESeq software (Bioconductor 3.12) was utilized to standardize gene count data across all samples and calculate fold changes in gene expression. The significance of differential expression was evaluated using negative binomial distribution tests. Differentially expressed genes (DEGs) were identified based on the criteria of “absolute fold change (FC) ≥ 1.5 and *p* < 0.05”. Finally, functional annotation and enrichment analysis of the identified DEGs were conducted using the Gene Ontology (GO; database link: http://geneontology.org/ (accessed on 28 October 2025)) and the Kyoto Encyclopedia of Genes and Genomes (KEGG; database link: https://www.kegg.jp/ (accessed on 28 October 2025), aiming to explore their biological functions and potential involvement in signaling pathways.

### 2.6. Detection of Reactive Oxygen Species

DCFH-DA fluorescent probe was used to detect intracellular ROS levels. HepG2 cell suspensions were seeded at a density of 1.5 × 10^5^ cells per well in a 6-well plate. After the cells reached 70~80% confluence, they were exposed to different concentrations of diiodoalkanes (25, 50, 100, and 200 µM) and 0.1% dimethyl sulfoxide (DMSO, solvent control) for 4 h and 24 h, respectively. After exposure, remove the culture medium from the wells. Add 10 µM DCFH-DA probe solution to each well. Incubate the cells in a 37 °C dark incubator for 30 min to ensure complete probe loading. After incubation, discard unloaded probe solution and gently wash cells three times with phosphate-buffered saline (PBS) to remove residual probe and minimize background fluorescence interference. Subsequently, observe and capture cellular fluorescence images using the ZOE™ Fluorescence Cell Imager (BIO-RAD, Hercules, CA, USA). Analyze the fluorescence images with Image-Pro Plus software (Medical Cybernetics, Inc., Albemarle, NC, USA, Version 6.0) to quantitatively calculate intracellular fluorescence intensity—the level of fluorescence indirectly reflects changes in intracellular ROS levels.

### 2.7. Determination of Catalase, Glutathione, and Cysteine

Commercially available assay kits were used to measure the levels of catalase (CAT), glutathione (GSH), and cysteine (Cys) in HepG2 cells following exposure to the toxicant. As described above, HepG2 cells were first treated with different concentrations of diiodoalkanes (including a 50 µM iodinated alkane group and a 0.1% DMSO solvent control group) for 24 h. Following treatment, cells were collected via trypsin digestion and gently washed 2~3 times with phosphate-buffered saline (PBS) to remove residual medium and digestion solution. Subsequently, intracellular levels of the three antioxidant molecules were measured using the CAT assay kit, GSH assay kit, and Cys assay kit, respectively. All procedures strictly followed the manufacturers’ instructions for each kit. After detection, the absorbance values corresponding to each indicator were recorded using a Spark^®^ 20M Multifunction Microplate Reader (Tecan, Switzerland). All assays were independently replicated three times to ensure the reliability of the experimental data.

### 2.8. Statistical Analysis

GraphPad Prism 9.0 software (San Diego, CA, USA) was used for graphing and statistical analysis. All experiments were independently replicated three times with at least three parallel samples. The data were presented as mean ± standard deviation (SD). Multiple comparisons between groups were analyzed by using one-way ANOVA and post hoc Tukey’s Test. The significance level of the data differences was determined by the *p*-values: *p*-value < 0.05 (*), *p*-value < 0.01 (**).

## 3. Results

### 3.1. Diiodoalkanes Affect the Cell Viability and Morphology of HepG2

**Figure 1:**
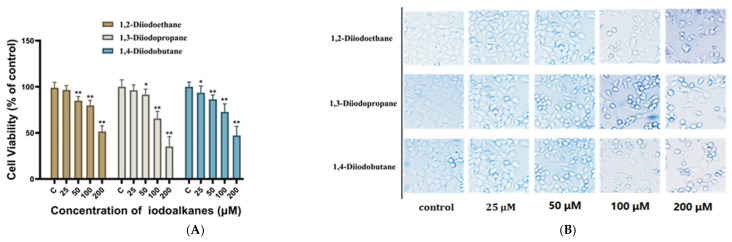
Effects of 1,2-diiodoethane, 1,3-diiodopropane and 1,4-diiodobutane exposure on HepG2. (**A**) The viability measured by the MTT assay. (**B**). The cell morphology observed by microscopic. (The control was treated with medium containing 0.1% DMSO (*v*/*v*), * *p* < 0.05, ** *p* < 0.01).

The MTT cell proliferation assay was employed to evaluate the effects of 24 h exposure to 1,2-diiodoethane, 1,3-diiodopropene, and 1,4-diiodoobutane on the proliferation ability of HepG2 cells. As shown in Figure 1A, compared with the control group, exposure to any of the three diiodoalkanes at concentrations of 25 µM or 50 µM led to a significant decrease in HepG2 cell viability (*p* < 0.01). These concentrations differ by less than 100-fold from those detected in actual surface water, suggesting potential ecological risks from diiodoalkanes in shale gas wastewater discharge. When the exposure concentration increased to 100 µM, all three diiodoalkanes significantly inhibited HepG2 cell viability (*p* < 0.05), reducing it to 79.9 ± 6.2%, 65.7 ± 7.5%, and 72.7 ± 8.4% of the control group, respectively. With a further concentration increase to 200 µM, cell viability continued to decrease significantly, reaching 51.6 ± 6.0%, 35.3 ± 10.3%, and 47.2 ± 9.5% of the control group, respectively. Quantitative analysis revealed the toxic potency of the three diiodoalkanes against HepG2 cells in the following order: 1,3-diiodopropane > 1,4-diiodobutane ≥ 1,2-diiodoethane.

The results of microscopic observations (Figure 1B) revealed significant morphological changes in HepG2 cells following 24 h exposure to the three diiodoalkanes. Compared with the control group, all treated groups exhibited typical toxicological characteristics, such as cell shrinkage, reduced cell numbers, and increased cellular debris. The extent of cell loss showed a positive correlation with diiodoalkanes concentration. At the high exposure concentration of 200 μM, specifically, a marked reduction in cell area was observed. The most pronounced decrease in cell number was observed in the 1,3-diiodopropane-treated group, followed by the 1,4-diiodobutane-treated group. This phenomenon aligns with the aforementioned reported toxicity ranking, further suggesting that the toxic effects of these three diiodoalkanes may be associated with their chemical structure.

### 3.2. Effects of Diiodoalkanes on Gene Expression in HepG2 Cells

**Figure 2:**
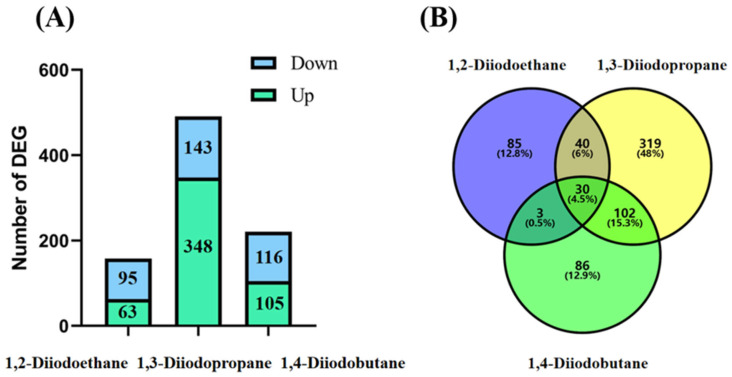
Analysis of differentially expressed genes (DEGs) after treatment of HepG2 cells with 1,2-diiodoethane, 1,3-diiodopropene, and 1,4-diiodobutane. (**A**) Number and distribution of DEGs in each group; (**B**) Venn diagram of DEGs.

To elucidate the molecular mechanisms underlying the cytotoxicity induced by diiodoalkanes on HepG2 cells, transcriptomic analysis was performed in this study to investigate differential gene expression profiles following exposure to 50 µM diiodoalkanes. The goal was to identify potential regulatory pathways mediating their toxic effects. Statistical results for differentially expressed genes (DEGs) are shown in Figure 2A. Compared with the control group, a total of 158 DEGs were identified in the 1,2-diiodoethane-treated group, including 63 upregulated genes and 95 downregulated genes; the number of DEGs in the 1,3-diiodopropane-treated group increased significantly to 491, comprising 348 upregulated and 143 downregulated genes; and 221 DEGs were detected in the 1,4-diiodobutane-treated group (105 upregulated and 116 downregulated). Figure 2B further presents the distribution characteristics of DEGs across the three treatment groups, clearly demonstrating the quantitative relationship between group-specific and shared DEGs. Specifically, 85 unique DEGs were identified in the 1,2-diiodoethane-exposed group, 319 in the 1,3-diiodopropane-exposed group, and 86 in the 1,4-diiodobutane-exposed group. Regarding shared DEGs, the 1,3-diiodopropane and 1,4-diiodobutane groups shared 132 DEGs, while the 1,2-diiodoethane group shared 70 and 33 DEGs with the 1,3-diiodopropane and 1,4-diiodobutane groups, respectively. These statistical data further indicate that the three diiodoalkanes have significantly different intensities of perturbation on the gene expression of HepG2 cells: 1,2-diiodoethane has a relatively weak regulatory effect on gene expression, while 1,3-diiodopropane has the most prominent perturbation effect on the cell transcriptome.

### 3.3. Gene Ontology Enrichment Analysis

**Figure 3:**
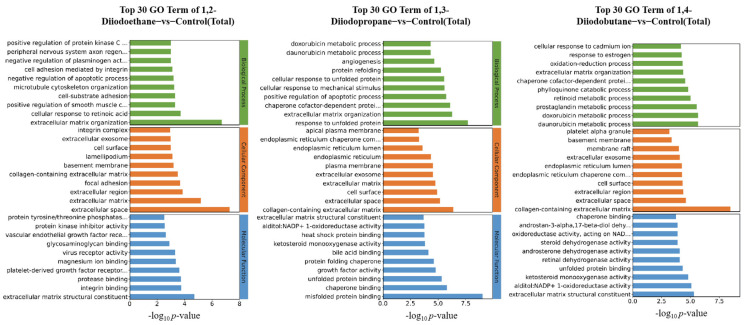
GO functional enrichment analysis of differentially expressed genes.

To clarify the biological functions associated with DEGs, Gene Ontology (GO) enrichment analysis was performed on the DEGs identified in this study. Figure 3 display the top 30 most significantly enriched biological function terms associated with DEGs in the 1,2-diiodoethane, 1,3-diiodopropane, and 1,4-diiodobutane treatment groups, respectively. These terms span three categories: biological process, cellular component, and molecular function, with the top 10 terms selected from each category. Analysis showed that nearly all significantly enriched GO terms were associated with the extracellular space and extracellular matrix. The core GO terms that are common to all three treatment groups and related to these extracellular components include: extracellular matrix organization (GO:0030198), extracellular space (GO:0005615), extracellular matrix (GO:0031012), extracellular exosome (GO:0070062), extracellular matrix structural constituent (GO:0005201), and collagen-containing extracellular matrix (GO:0062023). In the top GO enrichment results for the 1,3-diiodopropane and 1,4-diiodobutane exposure groups, both showed enrichment in GO terms related to endoplasmic reticulum function, specifically: endoplasmic reticulum lumen (GO:0005788), chaperone cofactor-dependent protein refolding (GO:0051085), and unfolded protein binding (GO:0051082). Additionally, GO terms enriched for 1,2-diiodoethane exposure, enriched GO terms were related to the negative regulation of the apoptotic process (GO:0043066), while for 1,3-diiodopropane exposure, terms related to the positive regulation of the apoptotic process (GO:0043065) were identified.

### 3.4. Kyoto Encyclopedia of Genes and Genomes Pathway Enrichment Analysis

**Figure 4:**
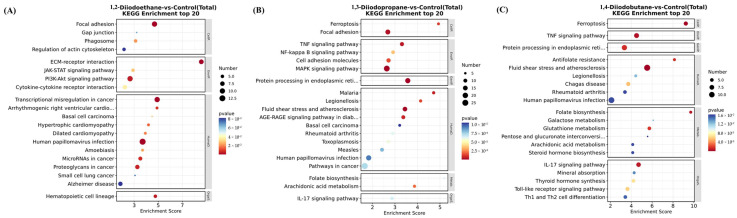
KEGG pathway analysis of differentially expressed genes.

To elucidate the potential molecular mechanisms underlying the effects of 1,2-diiodoethane, 1,3-diiodopropene, and 1,4-diiodobutane on HepG2 cells, this study further performed Kyoto Encyclopedia of Genes and Genomes (KEGG) pathway annotation and enrichment analysis on differentially expressed genes (DEGs). The top 20 signaling pathways significantly altered by the three diiodoalkanes were presented in Figure 4, respectively. Pathway analysis revealed that 1,2-diiodoethane primarily disrupted two key pathways: the ECM-receptor interaction pathway and the PI3K-Akt signaling pathway. In contrast, 1,3-diiodopropane and 1,4-diiodobutane shared partially overlapping disrupted pathways, specifically the ferroptosis pathway, TNF signaling pathway, IL-17 signaling pathway, and protein processing in the endoplasmic reticulum. Additionally, 1,3-diiodopropane uniquely regulated the MAPK signaling pathway. Previous studies have suggested that all the aforementioned disrupted pathways are directly or indirectly associated with oxidative stress [16,17,18,19,20]. These findings further suggest that 1,2-diiodoethane, 1,3-diiodopropane, and 1,4-diiodobutane significantly disrupt the homeostasis between oxidative stress-induced damage and antioxidant defense in HepG2 cells.

### 3.5. Effects of Diiodoalkanes on Reactive Oxygen Species in HepG2 Cells

**Figure 5:**
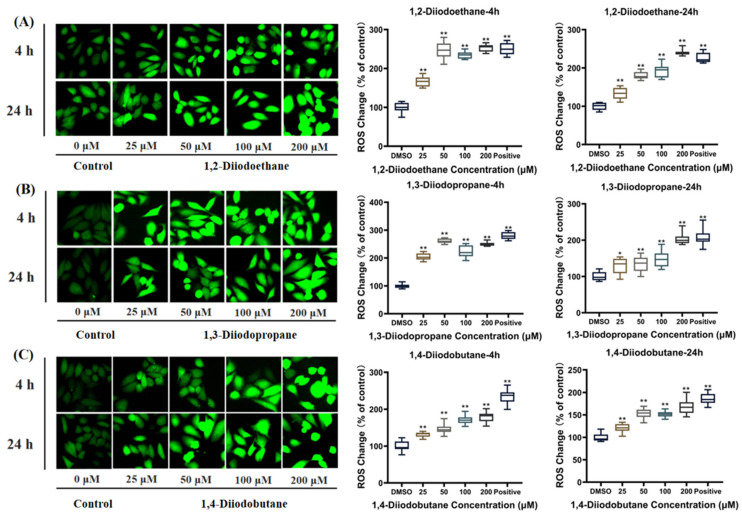
Effect of exposure to different iodinated alkanes on reactive oxygen species (ROS) generation in HepG2 cells. (**A**–**C**) show the fluorescence images and their corresponding quantification plots of ROS overproduction induced in cells by the 1,2-diiodoethane, 1,3-diiodopropene, and 1,4-diiodobutane groups, respectively. Values are the mean ± SD of three independent experiments. The control group was treated with medium containing 0.1% (*v*/*v*) DMSO. * *p* < 0.05, ** *p* < 0.01.

When cells undergo oxidative stress, the balance between pro-oxidative and antioxidant systems shifts toward oxidation, thereby inducing neutrophilic inflammatory infiltration, increased protease secretion, and the production of numerous oxidative intermediates [21,22]. Reactive oxygen species (ROS), as core markers of oxidative stress, directly reflect its severity. To investigate the effects of diiiodoalkanes on ROS levels in HepG2 cells under different exposure conditions, this study employed the DCFH-DA fluorescent probe assay. Five concentration gradients of 0, 25, 50, 100, and 200 µM were tested, with exposure durations of 4 h and 24 h, respectively. Results are shown in Figure 5. In the 4 h exposure group, the highest concentration (200 μM) of 1,2-diiodoethane, 1,3-diiodopropane, and 1,4-diiodobutane increased ROS levels in HepG2 cells to 253.2%, 250.2%, and 179.1% of the control group, respectively. In the 24 h exposure group, by contrast, ROS levels induced by these three compounds at the same concentration were 239.8%, 203.5%, and 168.3% of the control group, respectively. These results indicate that all three diiodoalkanes significantly induced an increase in ROS levels in HepG2 cells. The trend of ROS elevation was consistent across both 4 h and 24 h exposure groups, suggesting temporal stability in their oxidative stress-inducing effects.

### 3.6. Effects of Diiodoalkanes on Antioxidant Enzymes/Molecules in HepG2 Cells

**Figure 6:**
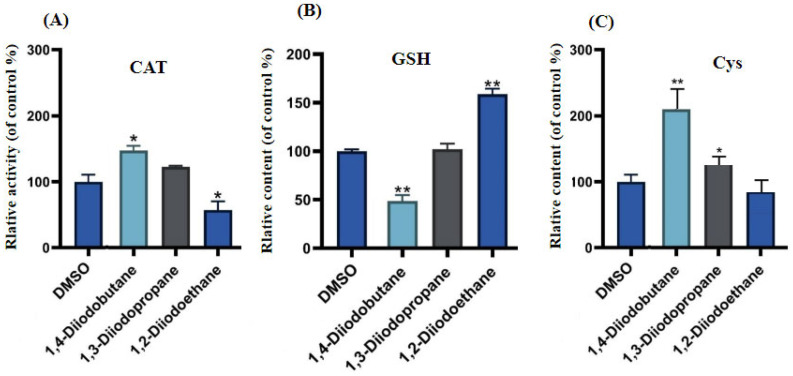
Effects of Diiodoalkanes on Antioxidant Enzymes/Molecules in HepG2 Cells. (**A**–**C**) show the relative activities or contents of catalase (CAT), glutathione (GSH), and cysteine (Cys) detected in HepG2 cells, respectively. Values are the mean ± SD of three independent experiments. The control group was treated with medium containing 0.1% (*v*/*v*) DMSO. * *p* < 0.05, ** *p* < 0.01.

Cells primarily rely on endogenous antioxidant systems to maintain ROS homeostasis, with cysteine (Cys), glutathione (GSH), and catalase (CAT) serving as core defense factors [23]. This study further examined antioxidant markers in HepG2 cells following 24 h exposure to three diiodoalkanes at a concentration of 50 µM. Compared with the control group, the three diiodoalkanes exhibited significant differences in their effects on CAT, GSH, and Cys in HepG2 cells. Regarding CAT activities (Figure 6A), exposure to 1,2-diiodoethane significantly reduced CAT activity to 57.1% of the control, while exposure to 1,4-diiodobutane significantly increased it to 147.3%. In contrast, exposure to 1,3-diiodopropane did not induce significant changes in CATactivities. For GSH levels (Figure 6B), 1,2-diiodoethane exposure caused an increase, whereas 1,4-diiodobutane exposure led tp a significant decrease. Similarly, 1,3-diiodopropane had no significant effect on GSH levels. With respect to Cys levels (Figure 6C), exposure to 1,2-diiodoethane did not result in significant changes, while both 1,3-diiodopropane and 1,4-diiodobutane exposure significantly elevated HepG2 intracellular Cys levels.

## 4. Discussions

Shale gas extraction not only releases pollutants into the surrounding environment—including fracturing additives and geogenic compounds—but also generates “unknown source” environmental transformation products, derived from both anticipated and unanticipated sources, in the process. Among these pollutants, organic iodides typically pose more significant health risks than chlorides or bromides; however, their presence and toxicological potential remain unclear. Previous studies have demonstrated that various di iodoalkanes exhibit cytotoxicity [11]. Their toxic effects not only exhibit significant time- and dose-dependence, but also vary in the intensity of toxicity based on the number of iodine substitutions in the molecule. Building on this, the present study further showed that three diiodoalkanes exerted clear effects on HepG2 cells at concentrations ranging from 25 to 200 μM: They significantly inhibited cell proliferation, induced cell morphological abnormalities, and even caused extensive cell death. These results further suggested the dose-dependent characteristics of the diiodoalkanes’ toxicity. This difference indicates that the toxicity of diiodoalkanes is closely related to their molecular structure. Mechanistically, the strong electron-withdrawing property of iodine atoms increases the molecular polarity, thereby promoting their binding to the intracellular enzyme systems. Ultimately, differences in molecular structure and iodine atom substitution positions determine the toxicity intensity of these pollutants.

Transcriptomic sequencing results revealed that the number of differentially expressed genes (DEGs) in HepG2 cells exposed to 1,2-diiodopropane, 1,3-diiodobutane, and 1,4-diiodoethane was 158, 491, and 221, respectively (Figure 2). Notably, the 1,3-diiodopropane-exposed group exhibited significantly more DEGs than the 1,2-diiodoethane and 1,4-diiodobutane groups, suggesting that 1,3-diiodopropane may exert a stronger molecular perturbation effect on HepG2 cells than the other two diiodoalkanes. This finding aligns with the established toxicity hierarchy of the three diiodoalkanes, further validating the correlation between molecular structure and toxic intensity. To elucidate the biological functions of identified DEGs, Gene Ontology (GO) and Kyoto Encyclopedia of Genes and Genomes (KEGG) enrichment analyses were conducted. The enriched GO terms were primarily associated with the extracellular space, extracellular matrix (ECM), and endoplasmic reticulum (ER). The extracellular space acts as an intermediary microenvironment for material exchange and signal transduction between cells and their surrounding environment. It is mainly composed of the ECM and extracellular fluids and also serves as a critical compartment for the diffusion and action of oxidative stress-related molecules, such as reactive oxygen species (ROS) and antioxidants. The ECM not only provides mechanical support and intercellular connectivity but also participates in intercellular signaling. It acts as a crucial binding site for cytokines and growth factors, and the maintenance of its structural and functional integrity is vital for sustaining cellular homeostasis [24]. When cells are subjected to adverse stimuli, the impaired function of the antioxidant defense system results in the overproduction of ROS, which disrupts the oxidant-antioxidant balance and induces oxidative stress. Oxidative stress not only directly damages ECM components (e.g., collagen and elastin) and matrix metalloproteinases (MMPs) but also downregulates the expression levels of ECM-related regulatory factors [25]. Notably, extracellular matrix remodeling is a key process jointly regulated by oxidative stress and DNA damage, exhibiting bidirectional interactions: oxidative stress/DNA damage induces ECM remodeling, while abnormal ECM remodeling conversely affects cellular oxidative stress status.

The ER serves as the primary site for protein folding within cells. The protein folding process mediated by it consumes ATP and involves redox reactions, consequently generating small amounts of ROS continuously. When cells are exposed to external toxins, the ER’s protein folding capacity becomes disrupted, leading to the accumulation of unfolded or misfolded proteins within its lumen. This induces endoplasmic reticulum stress (ERS), which further promotes the overproduction of ROS. Excessive ROS not only damages ER structure (e.g., by disrupting membrane integrity and oxidizing membrane phospholipids) but also oxidatively inactivates key functional molecules in the ER (e.g., molecular chaperones and foldases) [19]. This ultimately results in the total collapse of ER homeostasis, potentially inducing apoptosis.

For the 1,2-diiodoethane, 1,3-diiodopropane, and 1,4-diiodobutane exposure groups, the GO enrichment results all included entries related to the ECM. It is well established that oxidative stress can alter the metabolic processes of ECM components and the expression of related regulatory factors, leading to excessive accumulation or degradation of specific ECM components and the disruption of ECM homeostasis. Consistent with this mechanism, the aforementioned GO enrichment results imply that 1,3-diiodopropane and 1,4-diiodobutane may impair cell viability in HepG2 cells by exacerbating oxidative stress-induced damage and disrupting antioxidant function. Furthermore, KEGG enrichment analysis identified multiple signaling pathways related to the ECM and ER, nearly all of which have direct or indirect associations with oxidative stress. This finding further supports the notion that exposure to the aforementioned diiodoalkanes may induce cytotoxicity in HepG2 cells by perturbing the balance between oxidative stress-induced damage and antioxidant defense.

ROS are natural byproducts of normal cellular metabolic activity. Under physiological conditions, the production and clearance (quenching) of intracellular ROS maintain a dynamic equilibrium to ensure stable physiological functions. However, when cells are exposed to toxic stimuli, intracellular redox homeostasis is disrupted. The balance between ROS production and clearance shifts toward elevated ROS levels, leading to cellular dysfunction and the onset of oxidative stress [26]. The results of this study indicated that diiodoalkanes significantly induce increased ROS levels in HepG2 cells across the experimental exposure concentrations (25~200 μM) and exposure time points (4 h and 24 h). This elevation exhibits clear dose-dependent effects (Figure 4). Excessive intracellular ROS can trigger multiple outcomes, including oxidative damage to proteins and lipids, DNA damage, apoptosis, and inflammatory responses, potentially exerting significant adverse effects on HepG2 cells. Cells typically rely on their antioxidant systems to maintain ROS homeostasis. Therefore, to further investigate the regulatory effects of diiodoalkanes on the cellular antioxidant system, this study examined the levels of key enzymes and molecules closely associated with antioxidant function and redox homeostasis in HepG2 cells following 24 h exposure to the three diiodoalkanes. Results showed that: Compared with the control group, the 1,2-diiodoethane-exposed group exhibited significantly elevated GSH levels and markedly reduced CAT activity, with no significant change in Cys levels. The 1,3-diiodopropane-exposed group caused only a significant increase in cellular Cys levels. The 1,4-diiodobutane-exposed group led to a significant decrease in both CAT and Cys levels, along with a significant reduction in GSH levels. These findings indicated that diiodoalkanes can modulate cellular antioxidant systems by regulating key enzymes and molecules to counteract ROS accumulation, yet their regulatory patterns exhibit compound-specific structural dependencies.

## 5. Conclusions

This study focuses on “unknown-source” organic iodides generated during shale gas extraction—pollutants for which their occurrence, toxic effects, and mechanisms of toxicity remain unclear—and systematically investigates the toxic effects and underlying mechanisms of three typical diiodoalkanes on human hepatocellular carcinoma (HepG2) cells. Results show that at concentrations ranging from 25 to 200 μM, all three diiodoalkanes significantly inhibit HepG2 cell proliferation, induce cellular morphological abnormalities, and trigger extensive cell death; their toxicity exhibits pronounced time- and dose-dependent patterns, and 1,3-diiodopropane demonstrates the strongest toxicity. Transcriptomic analysis revealed that the number of differentially expressed genes (DEGs) in HepG2 cells treated with 1,2-diiodoethane, 1,3-diiodopropane, and 1,4-diiodobutane was 158, 491, and 221, respectively—with the 1,3-diiodopropane group exhibiting the highest DEG count—and this DEG profile perfectly aligns with the aforementioned toxicity hierarchy. Further Gene Ontology (GO) and Kyoto Encyclopedia of Genes and Genomes (KEGG) enrichment analyses showed that DEGs were primarily enriched in functional terms associated with the extracellular matrix (ECM) and endoplasmic reticulum (ER), suggesting that the toxic effects of diiodoalkanes may be linked to the disruption of cellular oxidant-antioxidant system homeostasis, and subsequent validation experiments suggested that diiodoalkanes exposure significantly increased reactive oxygen species (ROS) levels in HepG2 cells while markedly perturbing intracellular antioxidant molecules (glutathione [GSH], cysteine [Cys]) and the antioxidant enzyme catalase (CAT). However, this study has limitations, specifically as follows: First, the experimental model utilized HepG2 cells (a hepatocellular carcinoma cell line) rather than primary liver cells. Their physiological characteristics differ from those of normal liver cells, limiting the applicability of the findings for assessing health risks in real physiological scenarios. Second, the selected pollutants are volatile. Volatilization during experiments may have reduced the actual exposure concentration in the system, potentially leading to overestimated toxicity effect values that do not fully reflect the true toxicity profile under real environmental exposure conditions. Finally, due to technical limitations, we did not measure the actual concentration of pollutants taken up/utilized by the cells. Analysis was based solely on the initial exposure concentration. This hinders in-depth analysis of the metabolic pathways and mechanisms of action of pollutants within cells and also affects the precise interpretation of the dose–response relationship for toxic effects.

## Data Availability

The data that support the findings of this study are available from the corresponding author upon reasonable request.

## Supplementary Materials

The following supporting information can be downloaded at: https://www.mdpi.com/article/10.3390/toxics13110943/s1, Table S1: The physicochemical properties of the studied compounds. Table S2: Genes and their specific primers. Table S3: Preprocessing results of RNA-Sequencing Raw Data. Figure S1: Results of cluster analysis. Figure S2: Volcano plot showing the distribution of DEGs in HepG2 cells treated with 1,2-diiodoethane, 1,3-diiodopropane, and 1,4-diiodobutane. Red, green, and gray represent upregulation, downregulation, and no significant change in DEGs, respectively. A: 1,2-diiodoethane vs. control; B: 1,3-diiodopropane; C: 1,4-diiodobutane vs. control. Table S4: Comparison of gene expression between RT-qPCR and RNA-sequencing. Table S5: Dataset of all DEG.
