# Supplementary material for: Cytotoxicity of Typical Diiodoalkanes from Shale Gas Wastewater in HepG2 Cells
Source: Toxics. 2025 Oct 31;13(11):943. doi: 10.3390/toxics13110943 (PMC12656459; doi:10.3390/toxics13110943)
Supplement: Supplementary file 1 [file toxics-13-00943-s001.zip › toxics-3898396-supplementary.pdf]

Supporting Information

Cytotoxicity of Typical Diiodoalkanes from Shale Gas Wastewater in HepG2 Cells

Maoyuan Xu [a], Yusheng Wu [a], Yunmei Cai [b*], Ruijie Wang [a] and Guofa Ren [a*]
[a]Institute of Environmental pollution and health, School of Environmental and Chemical Engineering, Shanghai University, Shanghai, China, 200444
Environmental Protection Engineering, Foshan, Guangdong, China, 528216

* Correspondence: author: Dr. Guofa Ren, E-mail address: rgf2008@shu.edu.cn, Phone/fax: +86-21-66137753; Dr. Yunmei Cai, E-mail address: 18702030877@163.com, Phone/fax: +86-757-81773127

Methods，Qualty Assuarance and Quality Control

The physicochemical properties of studied compounds

Table S1 presents the physical and chemical properties of the compounds in study.

TableS1 The physicochemical properties of studied compounds

Compounds	Formula	CAS number	Molecular weight	Boiling point	Log P
1,2-Diiodoethane	$C_2H_4I_2$	624-73-7	282	206℃	1.86
1,3-Diiodopropane	$C_3H_6I_2$	627-31-6	298	169	3.02
1,4-Diiodobutane	$C_4H_8I_2$	628-21-7	310	152	2.64

RT-qPCR assay

We used cDNA as the template for real-time PCR to analyze the mRNA expression profile of twenty four genes after iodoalkanes exposing for 12 h. The sequences of all gene specific primers used in this study were indicated in Table 2. We used GAPDH as reference genes to normalize the transcript expression of target genes. The expression levels of target genes in Polymerase Chain Reaction (PCR) were detected by means of SYBR Green fluorescent dye. Based on the Ct value measured, the mRNA expression level of the target genes were determined using the 2-ΔΔCT method. Primer details are provided in Table S2.

Table S2 Genes and their specific primers

Gene symbol	Primer	Primer sequence (5′-3′)
SLC7A11	Forward Primer	GGTCCATTACCAGCTTTTGTACG
	Reverse Primer	AATGTAGCGTCCAAATGCCAG
SAT1	Forward Primer	ACCCGTGGATTGGCAAGTTAT
	Reverse Primer	TGCAACCTGGCTTAGATTCTTC
HO-1	Forward Primer	AAGACTGCGTTCCTGCTCAAC
	Reverse Primer	AAAGCCCTACAGCAACTGTCG
NCOA4	Forward Primer	GAGGTGTAGTGATGCACGGAG

	Reverse Primer	GACGGCTTATGCAACTGTGAA
FTL	Forward Primer	TCTTCATGCCCTGGGTTCTG
	Reverse Primer	GGTCCAAGGCTTGTTAGGATAGTT
SLC39A14	Forward Primer	TGCTCCTTCTCACTTCACCAC
	Reverse Primer	GACTTACTATACTCAGCTCTTACGC
CTSB	Forward Primer	CTACAGCCCGACCTACAAACA
	Reverse Primer	GAGCAGGAAGTCCGAATACAC

cDNA library construction and quality evaluation

The 0.1% DMSO solution served as the control group. The experimental groups were treated with 50 μM 1,2-diiodoethane, 1,3-diiodopropane, and 1,4-diiodobutane as experimental groups. Samples were collected 12 hours after treatment of HepG2 cells. Three replicates were set up for each sample, establishing a total of 12 cDNA libraries. Details are provided in Table S3. Raw sequencing reads of RNA samples contained low-quality bases that could interfere with subsequent analysis. To ensure data quality, low-quality bases were filtered out to obtain high-quality sequences (Clean Reads). The percentage of valid bases in sequencing reads exceeded 93.42%, with Q30 bases distributed between 91.8% and 93.078%. The GC content (total G and C bases) ranged from 50.06% to 50.62%, exhibiting uniform distribution and meeting quality control requirements for transcriptome sequencing.

Table S3 Preprocessing results of RNA-Sequencing Raw Data

Sample Name	RawReads (M)	RawBases (G)	CleanReads (M)	CleanBases (G)	ValidBases (%)	Q30 (%)	GC (%)
Control_1	47.74	7.16	47.11	6.75	94.25	92.3	50.62
Control_2	48.19	7.23	47.45	6.8	94.12	92.04	50.06
Control_3	47.39	7.11	46.63	6.71	94.37	91.8	50.25
S12_Diiodoethane_1	48.76	7.31	48.09	6.89	94.26	91.99	50.24
S12_Diiodoethane_2	46.67	7	45.92	6.61	94.45	92.7	50.11
S12_Diiodoethane_3	46.17	6.93	45.51	6.6	95.36	92.56	50.33
S13_Diiodopropane_1	47.08	7.06	46.36	6.66	94.24	92.8	50.36
S13_Diiodopropane_2	44.6	6.69	43.98	6.33	94.62	92.79	50.46
S13_Diiodopropane_3	50.66	7.6	49.94	7.12	93.64	93.07	50.27
S14_Diiodobutane_1	48.93	7.34	48.15	6.86	93.45	93.04	50.18
S14_Diiodobutane_2	48.3	7.25	47.51	6.77	93.42	92.82	50.16
S14_Diiodobutane_3	47.35	7.1	46.71	6.71	94.55	92.86	50.22

Cluster Analysis of samples

Cluster analysis of RNA sequencing results reveals the similarity between samples. As shown in Figure S1, all three replicate samples from each sample cluster closely together, indicating that replicate samples are in a similar state with minimal intra-sample variation. Furthermore, Figure S1 shows that the clustering results for the 1,2-diiodethane and 1,4-diiodobutane groups are similar, while the clustering results for the 1,3-diiodopropane group differ significantly from those of the Control group.

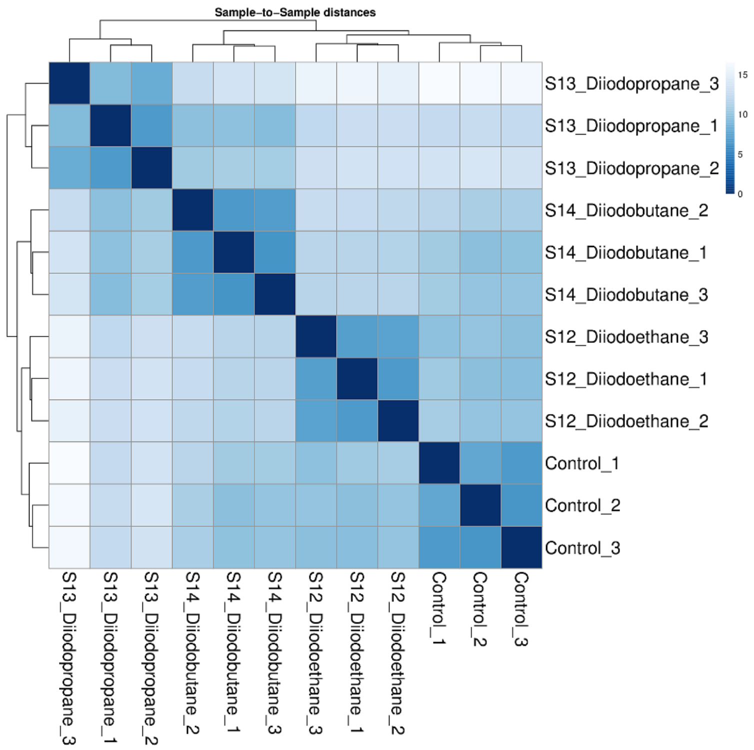

Figure S1 results of cluster analysis

Statistical Results for Differentially Expressed Genes

Figure S2 presents volcano plots for the differentially expressed genes (DEGs) across the three sample groups. The vertical dashed line indicates the FC=1.5 threshold, while the horizontal dashed line represents the p-value=0.05 threshold. Both plots demonstrate that compared to the 1,2-diiodoethane-exposed group, HepG2 cells exhibited greater gene disruption following treatment with 1,3-diiodopropane and 1,4-diiodobutane. 3-diiodopropane and 1,4-diiodobutane induced greater gene disruption than in the 1,2-diiodoethane group. Furthermore, the number of genes showing significant changes in HepG2 cells stimulated by 1,3-diiodopropane exposure exceeded that in the 1,4-diiodobutane group.

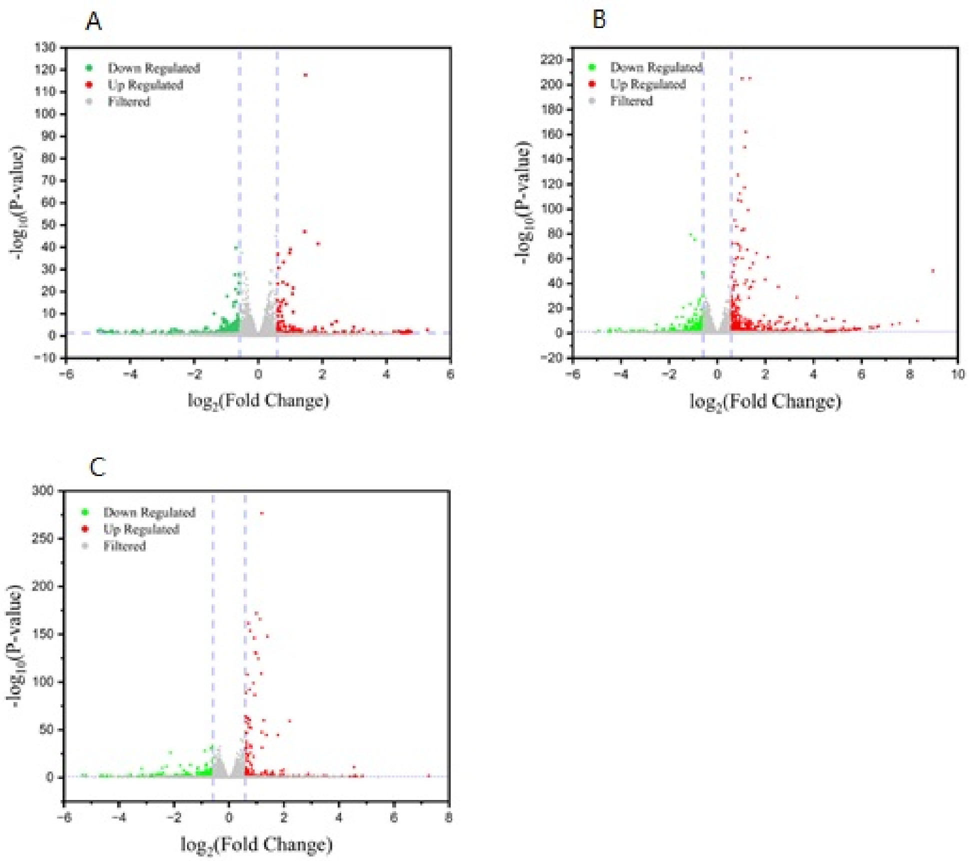

Figure S2 Volcano plot showing the distribution of DEGs in HepG2 cells treated with 1,2-diiodoethane, 1,3-diiodopropane, and 1,4-diiodobutane. Red, green and gray represent upregulation, downregulation, and no significant change in DEGs, respectively. A: 1,2-diiodoethane vs control； B: 1,3-diiodopropane； C: 1,4-diiodobutane vs control.

RT-qPCR validation

This study selected genes showing significant upregulation and no significant change to validate the reliability of transcriptomic sequencing data using RT-qPCR technology. The selected genes included SAT1, SLC7A11, HO-1, CTSB, FTL, SLC39A14, and NCOA4. Validation results are presented in Table S4. RT-qPCR-detected gene expression levels were largely consistent with RNA sequencing results, confirming the reliability of RNA-seq data for subsequent

in-depth analyses such as KEGG and GO enrichment studies.

Table S4 Comparison of gene expression between RT-qPCR and RNA-sequencing

Gene Name	Fold Change by RT-qPCR			Fold Change by RNA Sequencing		
	1,2-Diiodoethane	1,3-Diiodopropane	1,4-Diiodobutane	1,2-Diiodoethane	1,3-Diiodopropane	1,4-Diiodobutane
SAT1	1.55	2.36	1.57	1.87	1.63	1.74
SLC7A11	1.31	2.40	2.57	1.01	2.83	2.62
HO-1	1.90	2.90	2.10	0.86	5.79	2.40
CTSB	1.44	0.91	0.95	1.17	1.26	1.10
FTL	1.24	1.86	1.78	0.91	1.65	1.64
SLC39A14	1.40	0.93	0.97	1.24	1.15	0.98
NCOA4	2.36	1.11	1.14	0.96	0.92	0.95
